# Distinct pattern of enteric phospho-alpha-synuclein aggregates and gene expression profiles in patients with Parkinson’s disease

**DOI:** 10.1186/s40478-016-0408-2

**Published:** 2017-01-05

**Authors:** Martina Barrenschee, Dimitri Zorenkov, Martina Böttner, Christina Lange, François Cossais, Amelie Bernadette Scharf, Günther Deuschl, Susanne A. Schneider, Mark Ellrichmann, Annette Fritscher-Ravens, Thilo Wedel

**Affiliations:** 1 Institute of Anatomy, Kiel University, Kiel, Germany; 2 Department of Neurology, University Hospital Schleswig-Holstein, Campus Kiel, Kiel, Germany; 3 Experimental Endoscopy, Internal Medicine I, University Hospital Schleswig-Holstein, Campus Kiel, Kiel, Germany

**Keywords:** Parkinson’s disease, p-α-synuclein, Enteric nervous system, Gastrointestinal biopsies, Gene expression

## Abstract

Phosphorylated alpha-synuclein (p-α-syn) containing Lewy bodies (LBs) and Lewy neurites (LNs) are neuropathological hallmarks of Parkinson’s disease (PD) in the central nervous system (CNS). Since they have been also demonstrated in the enteric nervous system (ENS) of PD patients, the aim of the study was to analyze enteric p-α-syn positive aggregates and intestinal gene expression. Submucosal rectal biopsies were obtained from patients with PD and controls and processed for dual-label-immunohistochemistry for p-α-syn and PGP 9.5. p-α-syn positive aggregates in nerve fibers and neuronal somata were subjected to a morphometric analysis. mRNA expression of α-syn and dopaminergic, serotonergic, VIP (vaso intestinal peptide) ergic, cholinergic, muscarinergic neurotransmitter systems were investigated using qPCR. Frequency of p-α-syn positive nerve fibers was comparable between PD and controls. Although neuronal p-α-syn positive aggregates were detectable in both groups, total number and area of p-α-syn positive aggregates were increased in PD patients as was the number of small and large sized aggregates. Increased expression of dopamine receptor D1, VIP and serotonin receptor 3A was observed in PD patients, while serotonin receptor 4 and muscarinic receptor 3 (M3R) were downregulated. M3R expression correlated negative with the number of small sized p-α-syn positive aggregates. The findings strengthen the hypothesis that the CNS pathology of increased p-α-syn in PD also applies to the ENS, if elaborated morphometry is applied and give further insights in altered intestinal gene expression in PD. Although the mere presence of p-α-syn positive aggregates in the ENS should not be regarded as a criterion for PD diagnosis, elaborated morphometric analysis of p-α-syn positive aggregates in gastrointestinal biopsies could serve as a suitable tool for *in-vivo* diagnosis of PD.

**Electronic supplementary material:**

The online version of this article (doi:10.1186/s40478-016-0408-2) contains supplementary material, which is available to authorized users.

## Introduction

Parkinson’s disease (PD), a multicentric neurodegenerative disorder that’s progression extends to decades [31], is traditionally characterized by a loss of dopaminergic neurons in the central nervous system (CNS), mainly the brainstem, thus leading to characteristic cardinal motor symptoms such as bradykinesia, tremor, rigidity, and postural instability [43]. Degeneration of dopaminergic neurons and the presence of misfolded alpha-synuclein (α-syn) containing aggregates, namely Lewy bodies (LBs) and Lewy neurites (LNs), are primary pathological hallmarks of PD in the CNS. Within these aggregates phosphorylation at serin^129^ is the most dominant pathological α-syn form [3]. Recently, numerous studies have indicated that besides the CNS also the autonomic nervous system (ANS) is affected [36]. A wide spectrum of non-motor manifestations involving the urogenital and gastrointestinal (GI) system [32, 49] has been observed leading to impaired urinary function [5] and constipation [21] in patients with PD preceding the motor symptoms.

One main division of the ANS is represented by the enteric nervous system (ENS), a complex network integrated within the gut wall and organized in mainly two ganglionated nerve plexus, the myenteric and submucosal plexus [70]. Although the ENS works autonomously from the CNS [18], many morphological and functional properties of the ENS resemble the CNS rather than peripheral autonomic ganglia. Thus, the ENS is considered as “the little brain within the gut” [71], where it controls GI motility, mucosal blood flow, ion and water transport and resorption [24, 25]. Similar to the CNS, the functional integrity of the ENS depends on intact synaptic transmission and plasticity involving the synthesis, release and trafficking of a broad range of inhibitory and excitatory enteric neurotransmitters [23, 58].

Impairment of enteric neurotransmission is associated with a wide spectrum of functional GI diseases characterized by severe disturbances of GI motility [38] including constipation [21]. Constipation is also one of the leading premotor symptoms in patients with PD arguing for lesions within the ENS accompanying the progression of the disease [49]. Moreover, the ENS has been intensively discussed as a putative entry route of neurotoxins in PD suggesting that neuropathological processes spread via anatomically connected structures from the ENS to the substantia nigra and then further into other regions of the CNS [10, 47]. Thus, assessment of ENS pathology associated with PD might offer a twofold option: (1) to use the ENS as “diagnostic window into the CNS” [41] possibly allowing early *in vivo* diagnosis of PD and neuroprotective therapy, (2) to elucidate the pathogenetic mechanisms underlying the high prevalence of GI symptoms, in particular chronic constipation, in patients with PD.

Biopsies of the GI tract are the most promising peripheral tissue source to investigate the intestinal pathology linked to PD, as the tissue can be easily obtained by routine endoscopy and offers optimal conditions to study the ENS in living patients [40]. However, the specifity and usefulness of phosphorylated (p-α-syn) and native α-syn detection in colonic biopsies as a reliable biomarker of PD remains unclear due to highly variable results [2, 14, 17, 59] While some studies have regarded α-syn/p-α-syn in LB or LN as a discriminator between PD and controls [29, 39, 51, 52, 57], others have shown that expression of enteric α-syn/p-α-syn can also be observed in healthy subjects [2, 52, 61, 68]. In line with these findings, we could show previously that α-syn appears to be abundantly expressed in the ENS of healthy subjects and p-α-syn is not only detectable in patients with PD but also in controls in an age-dependent fashion [9].

Since p-α-syn is considered as the marker of choice to delineate pathological aggregates from physiological α-syn deposits, we aimed to further refine the assessment of p-α-syn in the ENS by morphometric quantification of phospho-serin^129^-α-synuclein (p-S^129^-α-syn) positive aggregates both in neuronal somata and nerve fibers in rectal biopsies of patients with PD and controls. Moreover, endoscopically retrieved biopsies allow to monitor gene expression profiles associated with intestinal pathological processes [16]. As PD is also associated with altered neurotransmitter systems [35, 54], we further analyzed the mRNA expression of α-syn and functionally relevant enteric neurotransmitter systems.

## Material and methods

### Patients

#### Patients with PD

Patients (*n* = 12, age range: 43–77 y, mean age: 65 y) were recruited from the Department of Neurology at the University Hospital Schleswig-Holstein, Campus Kiel. Diagnosis of PD was based on clinical examination by neurologists according to UK Parkinson’s Disease Society Brain Bank Clinical Criteria. Additional tests (e.g. cranial MRI, DAT-Scan, levodopa test) were carried out if required.

#### Controls

Control patients (*n* = 11, age range: 45–82 y, mean age: 66 y) were recruited from the Department of Internal Medicine I at the University Hospital Schleswig-Holstein, Campus Kiel, undergoing colonoscopy for colorectal cancer screening or GI symptoms without clinical evidence of neurological diseases. The study protocol received approval from the Local Ethics Committee of the Faculty of Medicine, Christian Albrecht’s University of Kiel, Germany (D455/10). From each patient a written informed consent was obtained.

### Clinical studies

#### Unified Parkinson disease rating scale part III (UPDRS-III)

All patients were assessed by the UPDRS-III [26]. For PD patients UPDRS-III was performed during ON-state. In addition, control patients underwent a detailed neurological examination to rule out PD symptoms and other neurodegenerative disorders.

#### Wexner constipation score

Symptoms of constipation were assessed by a validated constipation scoring system according to Wexner [1]. The test allows evaluating the severity of constipation.

### Retrieval and processing of rectal biopsies

**Fig. 1:**
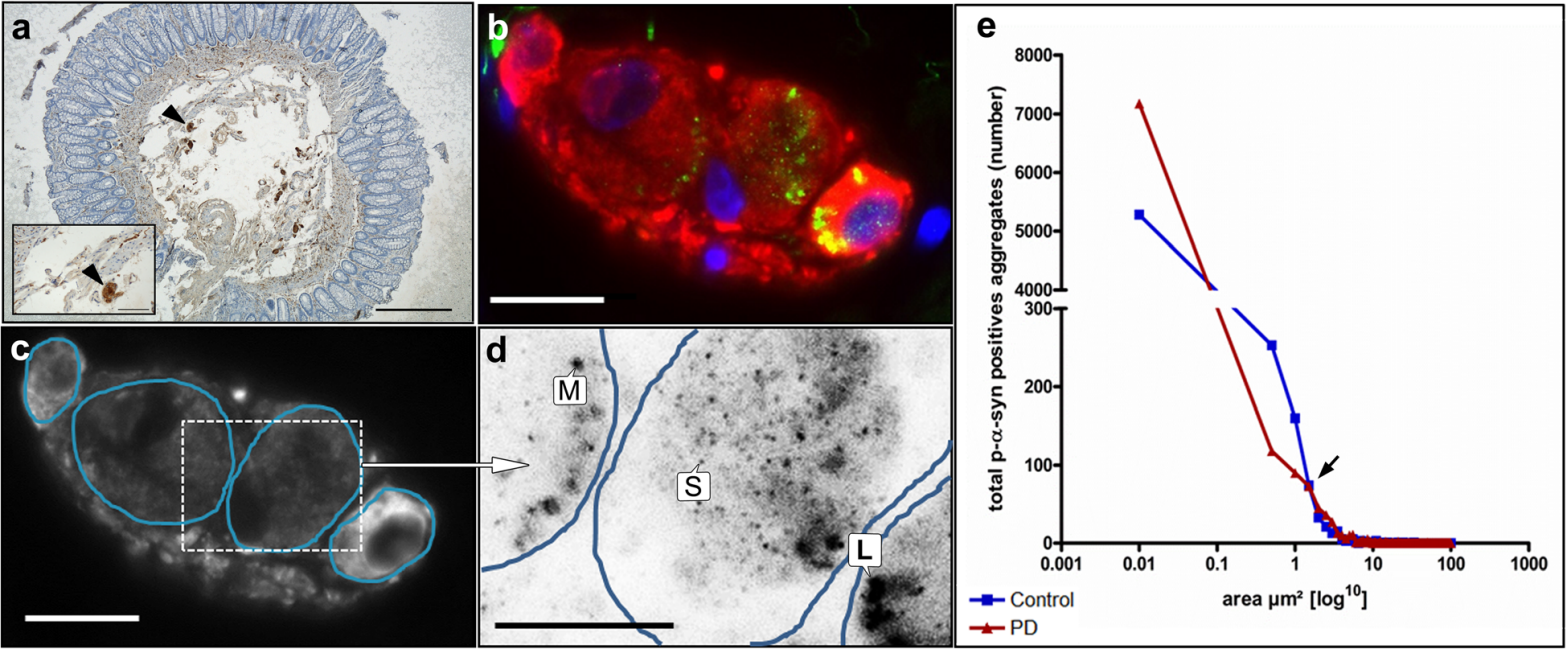
Methodical procedure of morphometric analysis of p-α-syn positive aggregates in neuronal somata of submucosal ganglia. **a** Screening of submucosal ganglia in orthogonal sections of a deep submucosal biopsy containing the mucosal and submucosal layer. Ganglia (*arrowhead* in (**a**) and inlet of **a**) and nerve fibers of the submucosal plexus were identified by conventional immunohistochemistry with the pan-neuronal marker PGP 9.5. **b** Dual-label immunohistochemistry with PGP 9.5 (*red*) and p-α-syn (*green*) of a submucosal ganglion. Number and area of neurons detected by the pan-neuronal marker PGP 9.5 were recorded (**c**, *blue circles*). **d** Insert from **c**. Total number and area of p-α-syn positive structures per neuron was calculated in photographs displayed in reverse grey-scale mode. p-α-syn positive aggregates were subdivided into three groups according to size (L = large size, M = medium size, S = small size). Nuclear counterstain with DAPI (*blue*), bars (**a**): 500 μm, inlet in (**a**) = 100 μm: (*b-d*): 20 μm. **e** To determine the threshold between medium and large size p-α-syn positive aggregates the crossing point of frequency distribution curves of p-α-syn positive aggregates in patients with PD (*blue squares*) and controls (*red triangles*) was defined (*black arrow*)

All patients underwent routine colonoscopy following a standard protocol. In each patient deep submucosal biopsies (*n* = 2) were obtained from the upper dorsal rectal wall and immediately fixed (4% paraformaldehyde in PBS) and stored at 4 °C until processing for immunohistochemistry and subsequent morphometric analysis. Additional biopsies (*n* = 4) for mRNA expressions studies were able to be retrieved from 12 patients with PD and 5 controls and immediately quick-frozen with liquid nitrogen and stored at −70 °C. For immunohistochemical studies biopsies were embedded into paraffin wax and cut orthogonally in serial sections (6 μm thickness) including the mucosa and submucosa. Screening for submucosal ganglia within the sections was performed by conventional immunohistochemistry using the pan-neuronal marker PGP 9.5 applied to every 7^th^ section (Fig. 1a). One adjacent section containing submucosal ganglia and nerve fibers was then used for subsequent morphometric analysis.

### Immunohistochemistry for PGP 9.5

Immunohistochemistry for the pan-neuronal marker PGP 9.5 was carried out as described previously [9]. Briefly, sections were incubated with 3% hydrogen peroxide, rinsed in TBS-buffer (TRIS-buffered saline; 10 mM TRIS, 50 mM NaCl, pH 7.4), incubated overnight with the polyclonal rabbit-anti-PGP 9.5 (PGP 9.5, 1:15000, Accurate Chemical & Scientific Corporation, Westbury, USA) diluted in antibody diluent (Invitrogen, Karlsruhe, Germany) and incubated for 45 min with biotinylated goat anti-rabbit IgG (1:400, DAKO, Hamburg, Germany). After washing three times with TBS, sections were incubated for 45 min with an avidin-biotin-complex (Vectastain ABC Standard, Vector Laboratories, Burlingame, USA) conjugated with horseradish peroxidase. 3, 3′-diaminobenzidine (DAKO, Hamburg, Germany) was used as substrate chromogen. Sections were counterstained with Meyer’s hematoxylin. Stained sections were analyzed by a light microscope (Nikon 6000, Nikon, Tokyo, Japan) coupled to a digital camera (Digital Sight, Nikon, Tokyo, Japan). Data acquisition was performed with NIS-Elements BR 3.2 software (Nikon, Tokyo, Japan). Omission of the primary or secondary antibody served as negative controls.

### Dual-label fluorescence immunohistochemistry for p-α-syn and PGP 9.5

Sections were pre-treated with citrate buffer (pH 6.0, 95 °C water bath) for 25 min followed by overnight incubation with a monoclonal mouse-anti-phospho-S^129^-α-syn antibody (Wako Clone: pSyn64, 1:1.000, Wako Chemicals GmbH, Neuss, Germany) in antibody diluent (Invitrogen, Karlsruhe, Germany) as primary antibody. The antibody was tested on human colonic and rectal carcinoma specimens and brainstem material from patients with Dementia with Lewy bodies in previous experiments [9]. After washing with TBS sections were incubated with goat anti-rabbit AlexaFluor488 antibody in antibody diluent (1:250, Invitrogen, Karlsruhe, Germany) as secondary antibody for 2 h. Sections were co-incubated with rabbit-anti-PGP 9.5 (PGP 9.5, 1:15000, Accurate Chemical & Scientific Corporation, Westbury, USA). Secondary antibody incubation was carried out with goat-anti-mouse AlexaFluor546 antibody (1:250; Invitrogen, Karlsruhe, Germany) for 2 h. Finally, all sections were stained with DAPI (Roche, Mannheim, Germany) to visualize cellular nuclei. Fluorescence signals were detected with a fluorescence microscope (Axiovert 200 M), (Zeiss, Göttingen, Germany), coupled to a digital monochrome camera (Axiocam) and captured with Axiovision software (Zeiss, Göttingen, Germany). Omission of the primary or secondary antibody served as negative controls.

### Quantitative morphometric analysis

#### Presence of p-α-syn positive structures

Sections from patients with PD and controls were assessed for the presence of p-α-syn positive structures. Screening of p-α-syn immunoreactive signals within biopsies was conducted separately for neuronal somata and nerve fibers. The portion of patients with PD and controls showing either p-α-syn positive neuronal somata or nerve fibers was calculated and expressed as percentage. In one control biopsy no ganglia could be found. Thus, this biopsy was only used for calculating the portion of p-α-syn positive nerve fibers.

#### Number and area of p-α-syn positive structures in neurons

All submucosal ganglia detected by the pan-neuronal marker PGP 9.5 were included in the morphometry. The ganglionic area as well as the area and number of neurons per ganglion were calculated (Fig. 1b,c). Mean neuronal area, mean ganglionic area and mean neuronal number per ganglionic area were calculated for controls and patients with PD (Additional file 1: Figure S2a-c). For each neuron, the total number and area of p-α-syn positive aggregates were recorded using the software ImageJ [60]. The total number of p-α-syn aggregates/neuron (Fig. 4a), the total area of p-α-syn positive aggregates/neuron (Fig. 4b), the total area of p-α-syn aggregates/neuronal area in % (Fig. 4c) and the total number of p-α-syn positive aggregates/neuronal area in μm (Fig. 4d) were calculated for controls and patients with PD.

#### Classification of small, medium and large size p-α-syn positive neuronal aggregates

Given the fact, that p-α-syn positive aggregates largely differed in size, they were further subdivided in small size, medium size and large size p-α-syn aggregates. Cut-off values were carried out as follows: Area of small size p-α-syn aggregates ≤ 0.1 μm^2^ was set to 0.06 μm^2^ corresponding to the mean of 10 calculated areas that exhibited a size ≤ 0.1 μm^2^ (Fig. 1d). For discrimination between medium and large size p-α-syn aggregates, frequency distribution curves were performed for both patients with PD and controls. The area was subdivided in intervals of 0.5 μm^2^ (range: 0–100 μm^2^) and the frequency of p-α-syn positive aggregates per interval was determined (Fig. 1e). The crossing point (arrow in e, 1.5 μm^2^) of both frequency distribution curves was defined as threshold between medium and large p-α-syn positive aggregates (Fig. 1e). Medium sized p-α-syn positive aggregates were thus defined as structures with size > 0.06 μm^2^ and ≤1.5 μm^2^, whereas large sized p-α-syn positive aggregates included all structures with size > 1.5 μm^2^ (Fig. 1d).

### Gene expression studies

#### RNA extraction and reverse transcription

Extraction of total RNA from quick-frozen biopsies were performed using the NucleoSpin total RNA/Protein isolation kit (Machery-Nagel, Düren, Germany) according to the manufacturer’s instructions. Total RNA was eluted in a volume of 60 μl H_2_O. Genomic DNA was digested for 15 min with 1.5 U of DNAse I (Sigma-Aldrich, Munich, Germany) at room temperature. Reverse transcription was carried out in a total volume of 30 μl containing 375 ng random hexamer primers (GE Healthcare, Freiburg, Germany), 0.5 mM dNTPs (Promega, Mannheim, Germany), 0.01 M DTT, 1 x reaction buffer, and 150 U Superscript II Reverse Transcriptase (Invitrogen, Karlsruhe, Germany). The annealing step was carried out at 25 °C for 10 min, elongation at 25 °C for 10 min, and denaturation at 70 °C for 15 min.

#### Real-time quantitative PCR (qPCR)

Gene expression was determined by real time quantitative polymerase chain reaction (qPCR) performed in 96 well plates in duplicate reactions, respectively. Each reaction (20 μl) contained 1 × qPCR Master Mix Plus (Eurogentec, Cologne, Germany), 900 nM primers, 225 nM hybridization probe and 2 μl of total cDNA. qPCR product accumulation was monitored by the ABI Prism 7700 Sequence Detection System (TaqMan, Applied Biosystems, CA, U.S.A.) over 45 cycles. Each cycle consisted of a denaturation phase (15 s at 95 °C) and a hybridization/elongation phase (1 min at 60 °C). Genes with primer and probes are listed in Table 1. The data were normalized to expression levels of the housekeeping gene hypoxanthine-guanine phosphoribosyltransferase (HPRT) expressed as fold increase and presented as mean ± SEM.

**Table 1:** Primer for target-genes used in quantitative-real-time PCR (qPCR). DRD1 primer was bought from ThermoFisher Scientific (Hs00265245_s1) as was DRD2 primer (MS00241436)

gene	forward primer	reverse primer	probe
HPRT	*5′-tgaacgtcttgctcgagatgtg-3′*	*5′-ccagcaggtcagcaaagaattt-3′*	*5′-tgggaggccatcacattgtagcc-3′*
SNCA	*5′-ccaaagagcaagtgacaaatgttg-3′*	*5′-agccagtggctgctgcaat-3′*	*5′-tgacgggtgtgacagcagtagccca-3′*
5HT3AR	*5-ggctggtgcacaagcaagac-3′*	*5-ggctggtgcacaagcaagac-3′*	*5-ctgcttggctgcgtcacctggttct-3′*
5HT4R	*5′-attccgggttgaacccttttc-3′*	*5′-aggtcttcggtagcgctcatc-3′*	*5′-acgtgccttcctcatcatcctct-3′*
M3R	*5′-cagctgcatacccaaaaccttt-3′*	*5′-tgaatgttttgttgcacagagcata-3′*	*5′-caacagcaccgtgaaccccgtgtg-3′*
VIP	*5′-aataaggcccagctccttgtg-3′*	*5′-cccaacctgagagcagaaggt-3′*	*5′-cttctcacagacttcggcatggc-3′*

### Statistical analysis

Data were analyzed by Mann–Whitney U test (Prism^TM^, GraphPad, Sand Diego, USA) and differences between groups were considered significant if *p* < 0.05 (*), *p* < 0.01 (**), or *p* < 0.001 (***). For correlation analysis Spearman’s rank correlation calculated by statistical software R 2.8.0 [53] was used and *p*-values were adjusted for multiple comparison using the method of Holm [30]. Clinical and morphometric data are documented as mean ± standard deviation.

## Results

### Clinical data

#### UPDRS-III

The clinical semi-quantitative UPDRS-III (motor symptom score, 0–108) yielded a mean score of 26.5 ± 16.1 in patients with PD and 0 in controls (Additional file 2: Figure S1a).

#### Wexner constipation score

The Wexner constipation score (constipation symptoms, 0–20) yielded a mean score of 4.1 ± 5 in patients with PD and 1.6 ± 4.2 in controls (Additional file 2: Figure S1b). Although the mean value was higher in patients with PD compared to controls, the difference was not statistically significant.

### Morphometric analysis of ganglia and neurons

In controls (*n* = 10) 51 ganglia with an overall number of 156 neurons could be detected, in patients with PD (*n* = 12) 48 ganglia with an overall number of 88 neurons could be detected. Mean neuronal area was not significant altered (248.2 ± 34.45 μm^2^) in PD patients compared to controls (282.8 ± 49.65 μm^2^) (Additional file 1: Figure S2a). Mean ganglionic area of PD patients (1264 ± 188.4 μm^2^) was slightly but not significantly decreased compared to controls (855.9 ± 143.7 μm^2^). (Additional file 1: Figure S2b).

Mean neuronal number per ganglionic area of patients with PD was not significant altered (0.002698 ± 0.0004841 number/μm^2^ ganglion for controls; 0.002814 ± 0.0004843 number/μm^2^ ganglion for patients with PD) (Additional file 1: Figure S2c).

### Pattern and detection rate of p-α-syn immunoreactivity

#### Staining pattern of p-α-syn positive neuronal somata and nerve fibers

**Fig. 2:**
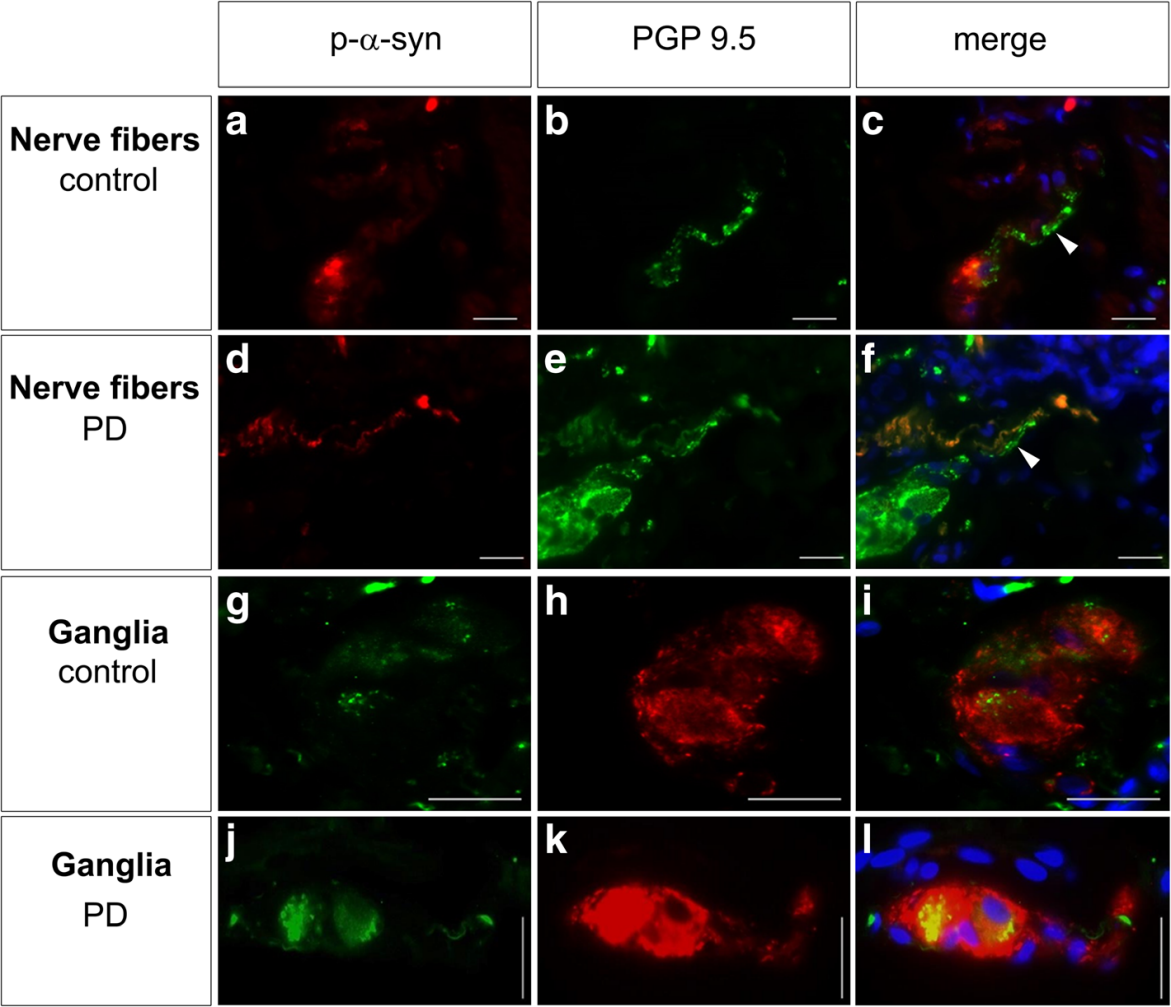
Dual-label fluorescence immunohistochemistry of p-α-syn and PGP 9.5 in submucosal ganglia and nerve fibers. p-α-syn immunoreactive signals (*red*) were detected in nerve fibers visualized by PGP 9.5 (*green*) as inhomogeneous and punctate staining pattern in both controls (**a**-**c**) and patients with PD (**d**-**f**). However, not all of the PGP 9.5 positive nerve fibers were also immunoreactive for p-α-syn (*arrowheads* in **c** and **f**). Within submucosal ganglia visualized by PGP 9.5 (**red**) p-α-syn immunoreactive signals (*green*) displayed aggregates of different sizes mainly detected in neuronal somata of both controls (**g**-**i**) and patients with PD (**j**-**l**). No p-α-syn positive staining was found in cell nuclei, but slight punctate signals were discernible in the ganglionic neuropil. Nuclear counterstain with DAPI (*blue*), bars: 20 μm

p-α-syn immunoreactive signals were detected in nerve fibers as discontinuous or punctate staining pattern in both patients with PD and controls (Fig. 2a-f). However, not all of the PGP 9.5 positive nerve fibers were also immunoreactive for p-α-syn, and in some instances immunoreactivities for p-α-syn and PGP 9.5 did not completely merge (Fig. 2c,f). Within submucosal ganglia p-α-syn immunoreactive signals were mainly detected in neuronal somata of both patients with PD and controls exhibiting aggregates of different sizes (Fig. 2g-l). No p-α-syn positive staining was found in cell nuclei, however slight punctate signals were discernible in the ganglionic neuropil.

#### Detection rate of p-α-syn positive neuronal somata and nerve fibers

**Fig. 3:**
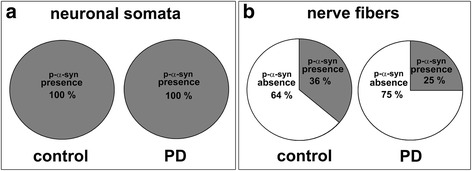
Presence of p-α-syn positive neuronal somata and nerve fibers. While p-α-syn positive neuronal somata were found in all submucosal biopsies of patients with PD and controls (**a**), p-α-syn positive nerve fibers were detected in 4 (36%) out of 11 control individuals and 3 (25%) out of 12 patients with PD, respectively (**b**)

In both patients with PD and controls p-α-syn immunoreactive signals were readily detectable within the submucosal plexus. While p-α-syn positive aggregates in neuronal somata could be identified in each biopsy obtained from healthy subjects as well as patients with PD (Fig. 3a), p-α-syn positive nerve fibers were not consistently detectable. In controls, 4 (36%) out of 11 individuals showed positive p-α-syn signals in nerve fibers, in the PD group, only 3 (25%) out of 12 patients displayed p-α-syn positive nerve fibers (Fig. 3b).

### Quantitative morphometric analysis of p-α-syn positive aggregates

As p-α-syn immunoreactive signals were detected in submucosal neurons in both controls and patients with PD (Fig. 2), we performed quantitative morphometric analysis to further test whether p-α-syn immunoreactive aggregates differed between groups in regards to number, area and size.

#### Number and area of p-α-syn positive aggregates

**Fig. 4:**
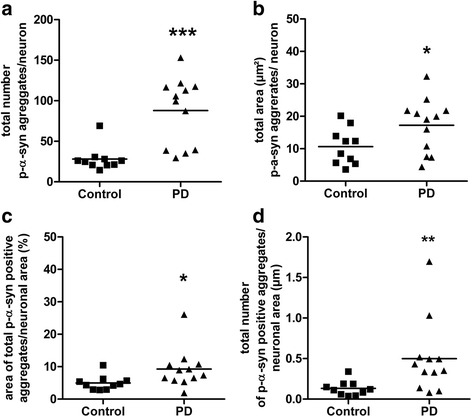
Number and area of p-α-syn positive aggregates. The total number of p-α-syn positive aggregates per neuron (**a**) and the area of p- α -syn positive aggregates per neuron (**b**) are significantly increased in patients with PD (*n* = 12) compared to controls (*n* = 10). The percentage of p-α-syn positive aggregates per neuron was significant increased in patients with PD (**c**) as was the total number of p-α-syn positive aggregates normalized to the neuronal area (**d**). Data are presented as mean ± SEM; *p < 0.05 vs control; ***, *p* < 0.001 vs control

The total number of p-α-syn positive aggregates per neuron was signficantly higher in patients with PD (88 ± 42 p-α-syn positive aggregates/neuron) compared to controls (28 ± 15 p-α-syn positive aggregates/neuron) (Fig. 4a). Patients with PD also showed a significantly increased total area occupied by p-α-syn positive aggregates within neurons (17.40 ± 8.06 μm^2^ p-α-syn positive aggregates/neuron) compared to controls (10.83 ± 5.77 μm^2^ p-α-syn positive aggregates/neuron) (Fig. 4b). When normalized to the neuronal area, the total area of p-α-syn positive aggregates was also significantly increased in patients with PD (9.261 ± 1.733%) compared to controls (4.958 ± 0.717%) (Fig. 4c). Also total number of p-α-syn positive aggregates per neuronal area was significantly increased in patients with PD (0.5553 ± 0.1450) when compared to controls (0.2147 ± 0.03874) (Fig. 4d).

#### Distribution of p-α − syn positive aggregates according to size

**Fig. 5:**
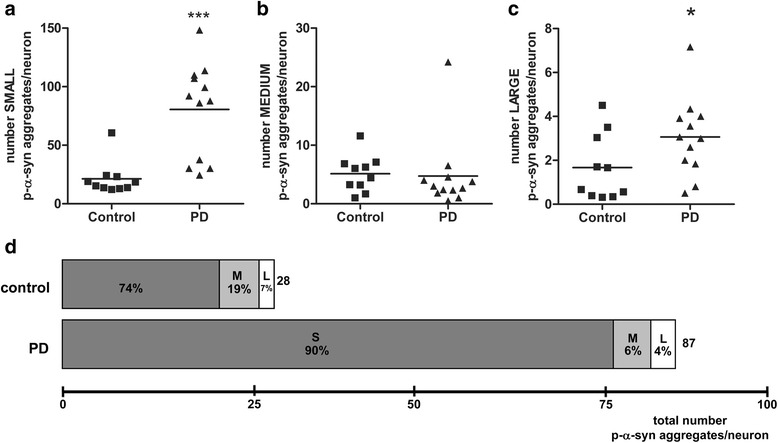
Distribution of p-α-syn positive aggregates according to size. Whereas for medium sized p-α-syn positive aggregates no significant differences were found between patients with PD (*n* = 12) and controls (*n* = 10) (**b**), patients with PD showed increased numbers of both small sized (**a**) and large sized (**c**) p-α-syn positive aggregates compared to controls. **d** Total number of p-α-syn positive aggregates per neuron was higher in patients with PD compared to controls due to the high amount of small sized p-α-syn positive aggregates. The relative portion of small (S) sized p-α-syn aggregates was higher in patients with PD, while the relative portion of medium (M) and large (L) sized p-α-syn aggregates were higher in controls (**d**). Data are presented as mean ± SEM; *p < 0.05 vs control; ***, *p* < 0.001

p-α − syn positive aggregates were subdivided according to size into small, medium and large size p-α − syn aggregates and quantified for both groups. Whereas for medium size p-α − syn aggregates no significant difference was found between patients with PD (3.16 ± 1.70) and controls (5.34 ± 3.11) (Fig. 5b), patients with PD showed increased number of both small size (80.52 ± 44.47) and large size (3.20 ± 1.76) p-α − syn aggregates per neuron compared to controls (21.33 ± 14.40, 1.67 ± 1.51) (Fig. 5a,c). Moreover, calculation of the relative distribution of p-α − syn positive aggregates (Fig. 5d) revealed a different distributional pattern between patients with PD and controls. While patients with PD showed 90% ± 7% small size 6% ± 5.5% medium sized and 4% ± 4.9% large size p-α − syn aggregates, controls displayed 74% ± 13%, 19% ± 11% and 7% ± 6.7% p-α − syn aggregates, respectively.

#### Clinicopathological correlation analysis in patients with PD

Correlation analysis of results obtained from the UPDRS-III scale and Wexner Score showed no correlation between the severity of PD symptoms and of constipation symptoms (Spearman correlation coefficient (r_s_) = −0.249, adjusted *p* value = 1). Also no correlation between histological findings and UPDRS-III results was found (total area of p-α − syn aggregates/neuron, r_s_ = 0.252, *p* = 1; total number of p-α − syn aggregates/neuron, r_s_ = −0.148, *p* = 1; large size p-α − syn aggregates/neuron, r_s_ = 0.329, *p* = 1; medium size p-α − syn aggregates/neuron, r_s_ = −0.192, *p* = 1; small size p-α − syn aggregates/neuron. r_s_ = −0.148, *p* = 1). Finally, no correlation was observed between histological findings and Wexner Score results (total area of p-α − syn aggregates/neuron, r_s_ = 0.252, *p* = 1; total number of p-α − syn aggregates/neuron, r_s_ = −0.148, *p* = 1; large size p-α − syn aggregates/neuron, r_s_ = 0.329, *p* = 1; medium size p-α − syn aggregates/neuron, r_s_ = −0.192, *p* = 1; small size p-α − syn aggregates/neuron. r_s_ = −0.148, *p* = 1).

### Gene expression analysis

**Fig. 6:**
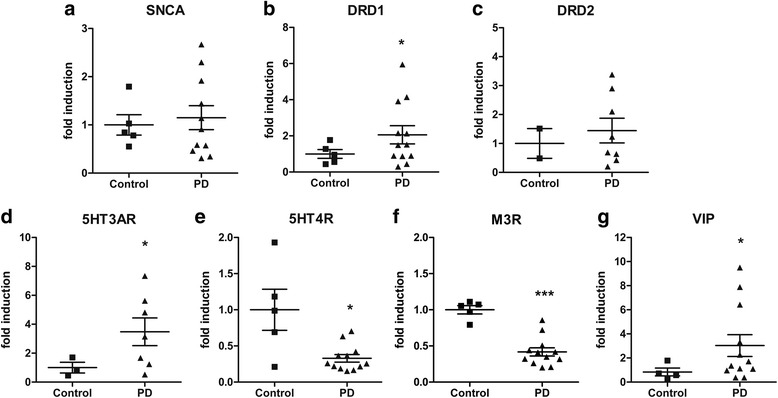
Gene expression analysis of synuclein and enteric neurotransmitter systems. mRNA expression in submucosal biopsies of patients with PD (*n* = 8-12) and controls (*n* = 2-5) showed no alteration in synuclein (SNCA, **a**) and D2 subtype of dopamine receptor (DRD2, **c**), whereas significant upregulation of D1 subtype of dopamine receptor (DRD1, **b**), subunit A of type 3 receptor for 5-hydroxytryptamine (serotonin) (5HT3AR, **d**), vasoactive intestinal peptide (VIP, **g**) was found in patients with PD. mRNA expression of 5-hydroxytryptamine (serotonin) receptor 4 (5HT4R, **e**) and muscarinic acetylcholine receptor M3 (M3R, **f**) were significantly down-regulated in patients with PD. Data were normalized to the mRNA expression of the house-keeping gene HPRT and presented as mean ± SEM and expressed as fold induction.*, *p* < 0.05 vs control; **, *p* < 0.01 vs control; ***, *p* < 0.001 vs control

mRNA expression was analyzed for α − syn (SNCA) and selected genes of enteric neurotransmitter systems including dopaminergic, serotonergic, and cholinergic receptors, as well as the neurotransmitter vasoactive intestinal peptide (VIP). SNCA showed no difference in mRNA expression between patients with PD and controls (Fig. 6a). While mRNA expression of dopamine receptor D1 subtype (DRD1) exhibited a significant increase in patients with PD (Fig. 6b), mRNA expression of D2 subtype (DRD2) remained unaltered (Fig. 6c). mRNA expression of the subunit A of the type 3 receptor for 5-hydroxytryptamine (serotonin) (5HT3AR) was significantly up-regulated in patients with PD (Fig. 6d), whereas 5-hydroxytryptamine (serotonin) receptor 4 (5HT4R) was significantly down-regulated (Fig. 6e). mRNA expression of the muscarinic acetylcholine receptor M3 (M3R) was significantly decreased (Fig. 6f), mRNA expression of VIP was significant up-regulated in patients with PD (Fig. 6g).

### Correlation analysis between histological findings and gene expression data

**Fig. 7:**
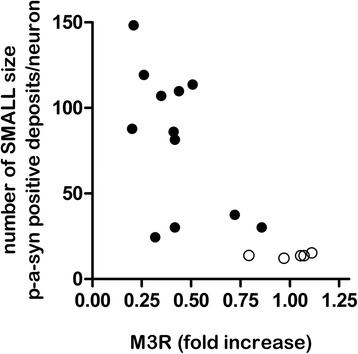
Correlation analysis of p-α-syn positive aggregates and gene expression profiles. Correlation analysis of p-α-syn positive aggregates (total number, total area, number of small, medium and large sized aggregates) and gene expression data identified a negative correlation between small sized p-α-syn positive aggregates and gene expression levels of the muscarinic acetylcholine receptor M3 (M3R), with a Spearman correlation coefficient of −0.72 and adjusted *p*-value of < 0.0001. Black filled circles showed data points of the PD group; white circles showed data points of the control group

Analysis of correlation between p-α-syn positive aggregates (total number, total area, number of small, medium and large size p-α − syn aggregates) and gene expression data showed no correlation except for one negative correlation between the number of small size p-α-syn positive aggregates and gene expression levels of the muscarinic acetylcholine receptor M3 (M3R), independent of the different groups (Spearman correlation coefficient (r_s_): −0.72, adjusted *p*-value < 0.0001) (Fig. 7). Correlation between the number of small size p-α-syn positive aggregates and gene expression levels of the muscarinic acetylcholine receptor M3 (M3R) dependent on the groups showed no significant correlation in the PD group (r_s_ = −0.356, *p* = 1) and control group (r_s_ = 0.62, *p* = 1).

## Discussion

### Positive p-α-syn staining in patients with PD and controls

Several *in vivo* studies of patients with PD using α-syn and/or p-α-syn immunohistochemistry applied to GI tract biopsies reported different results in positive staining rates (for reviews see [2, 59, 64]) with a range from 5 to 100% for PD and from 0 to 100% for controls. The high variability of these staining rates might be explained by the different GI tract sites studied and technical work-up of biopsies (e.g. tissue handling, fixation, antigen retrieval, primary antibodies). In most of the studies p-α-syn immunoreactivity was not observed in healthy controls suggesting p-α-syn detection as an useful and specific tool for identifying patients with PD [29, 52, 57].

However, a few studies [9, 52, 68] reported the presence of p-α-syn in colonic biopsies also in healthy controls. By using conventional immunohistochemistry and the highly specific PET-blot technique *Visanji et al.* revealed p-α-syn positive aggregates in mucosal biopsies of all healthy individuals investigated [68]. The authors concluded that enteric p-α-syn immunoreactivity is not an appropiate discriminator between healthy controls and patients with PD. In line with these findings, we could also observe positive p-α-syn staining not only in patients with PD but also in all biopsies obtained from controls. By using fluorescence p-α-syn immunohistochemistry applied to rectal wholemount biopsies, Pouclet *et al*. found mild somatic labelling of submucosal neurons both in patients with PD and controls and described dot-like pathological inclusions in p-α-syn positive structures of patients with PD compared to controls. Most of these structures are immunoreactive for the neuronal marker neurofilament 200 kDa, but some of the inclusions do not display neurofilament 200 kDa immunoreactivity, even though the authors find, that their morphology is highly suggestive of Lewy neurites [52]. Although our findings of p-α-syn positive neuronal and non-neuronal submucosal structures (somatic labeling and dot-like pathological inclusions in p-α-syn positive nerve fibers, see Fig. 2) matched that of Pouclet and colleagues, we did not find a significant difference in p-α-syn staining rates in patients with PD, as p-a-syn staining rates for nerve fibers in the present study were 25% for patients with PD and 36% for controls (Fig. 3b). Since both studies used the same antibody, the discrepancies might be related to technical aspects (e.g. unsectioned wholemount tissue vs. tissue sections, different antibody dilutions) or due to different biopsy sampling sites in the GIT (colon vs. rectum).

In summary, given that p-α-syn was readily detectable within the submucosal plexus likewise in patients with PD and controls, the present data suggest that the mere presence of p-α-syn cannot be regarded as specific for PD. For that reason we have carried out a refined quantitative morphometric analysis of the amount and pattern of p-α-syn aggregates aiming at better discriminating between patients with PD and healthy subjects.

### Increased number and area of p-α-syn positive aggregates in patients with PD

Morphological heterogeneity of LB-like structures has been already observed in the CNS appearing as either diffuse granular pleomorphic structures of variable shape and size or intraneuritic dot-like structures [7, 20]. In the ENS *Gold et al.* [27], have found also different staining pattern ranging from light, diffuse or punctate α-syn positive labeling limited to nerve terminals to large clustering of rings and puncta in some but not all myenteric somata. Consistent with these findings we could also describe both homogeneous and granular p-α-syn labeling in myenteric and submucosal ganglia [9].

Although additional parameters to characterize p-α-syn aggregates in more detail (e.g. distributional pattern) appear to be required, so far this quantitative morphometric approach has not been carried out previously. In fact, the assessment of the total number and area of p-α-syn positive structures per neuron revealed that patients with PD showed a significant increase of both parameters compared to controls. The overlap of values between both groups was lower for the number of p-α-syn positive structures per neuron suggesting that this parameter discriminates even better between patients with PD and controls than the area of p-α-syn positive structures.

### Distinct distributional pattern of p-α-syn aggregates according to size in patients with PD

Further subdivision of neuronal p-α-syn aggregates according to size revealed a significant increase of small and large sized p-α-syn aggregates in patients with PD compared to controls, while medium sized p-α-syn aggregates were equally distributed in both groups. The best discriminator was the number of small sized p-α-syn aggregates - only one third of patients with PD fell within the range of controls, whereas two thirds showed values well above the control group.

The (patho-) physiological mechanisms leading to the formation of differently sized p-α-syn aggregates observed within the ENS remains unclear. The well-known morphological heterogeneity of LBs within the CNS has fed speculations that the spectrum of α-syn inclusions could represent different stages of LB development [28, 55]. *Gomez-Tortosa et al.* hypothesized that clouds of rich α-syn containing structures could represent very early stages of LB pathology. Such lesions would progress to more compact and better-defined α-syn aggregates and some of these inclusions could be recognized as pale bodies in H&E stain considered as precursors of LBs [69], which in turn condense to classical LBs. As p-α-syn is a subcomponent of LBs [62], it might be assumed that the differently sized p-α-syn positive aggregates observed within enteric ganglia could represent different LB development stages ranging from small sized granula to the assembly and fusion into larger aggregates.

It is of note that although the number of small and large sized p-α-syn aggregates was significant increased in patients with PD, all three sizes of p-α-syn aggregates were also observed in controls. This suggests that p-α-syn aggregation in the gut is a physiological process, but may be dysregulated and increased in patients with PD. The following observations strengthen this hypothesis: (1) Both p-α-syn and α-syn are physiologically expressed in the human [8, 9] and rat ENS [50]. (2) α-syn aggregates are primarily cytoplasmic [63] and although most of the studies observed physiological cytoplasmic α-syn as monomer [12], some studies indicate that α-syn can assemble also physiologically as oligomers [6, 37] and upon membrane binding as multimers [11]. (3) Environmental toxins (e.g. rotenone) trigger PD-like progression by promoting α-syn release from enteric neurons, uptake by presynaptic neurites and retrograde transport to and accumulation in neuronal somata. [48]. Thus, it is suggestive that increased p-α-syn positive aggregates in enteric neurons, in particular the small sized type, in PD patients reflect the inital steps of an enhanced α-syn release upon external insults.

### Gene expression of α-syn

Interestingly, no difference in the SNCA mRNA expression between PD and controls was observed, even though total number and area of p-α-syn positive aggregates were significantly increased in PD patients. However, also in the CNS PD, particularly the sporadic form, is not consistently associated with increased mRNA levels of SNCA [15, 67]. In addition, some studies showed that LB formation is also not associated with an increase in SNCA mRNA expression [34]. In contrast to that, α-syn secretion normally is associated with an increase in SNCA mRNA expression [44]. This strengthen the idea that our observed increase in neuronal p-α-syn positive aggregates is the product of rather a disturbed α-syn aggregation process than an increase in α-syn secretion.

### Altered gene expression of neurotransmitter systems

In line with our findings a decrease of 5-HT4 receptors in the tunica muscularis has also been observed in a mouse-model of PD [74]*.* Therefore, the dysregulated 5-HT4 and 5-HT3 receptor mRNA expression observed in our study might reflect mechanisms underlying colonic dysmotility in PD. DA is known to inhibit GI motility via D1 receptors [72] and the M3 receptor is an important subtype mediating the contraction of intestinal smooth muscle [66]. Thus, both the significant upregulation of D1 receptor mRNA and downregulation of M3 receptor mRNA may further contribute to constipation symptoms in patients with PD. However, the upregulation of VIP mRNA does not fit into this concept, as this neuropeptide is downregulated in patients with idiopathic constipation [46]. Generally, interpretation of enteric neurotransmitter disturbances observed within the mucosal/submucosal layer retrieved by biopsies should be made carefully in regard to its effects on GI motility, as GI motility is primary driven by the myenteric plexus. Full-thickness biopsies including all enteric nerve plexus would provide optimized conditions to study these aspects. In addition in a previous study Annerino *et al.* investigated the relative abundance of NO, VIP or TH neurons between patients with PD and controls, by counting nitric oxide synthase (NOS), VIP, or TH fluorescence labelled neurons [4]. The authors reported no difference in relative abundance of NO, VIP, or TH neurons in myenteric plexus of any GI segment of patients with PD. Thus, neuropathology in myenteric neurons as causative factor for PD-related GI dysmotility is still under discussion.

Consistent with our results, reduced mucosal expression of 5-HT4 receptor and increased mucosal expression of 5-HT3A receptors was also observed in a mouse model of experimental colitis [45]. Additionally, 5-HT4 receptor activation is linked to anti-inflammatory effects in the GI system [76], and increased mRNA expression of VIP was reported in moderately inflamed mucosal epithelium [33]. These data suggest a link between PD and inflammatory processes at the level of the GI tract, as proposed by *Devos et al.*, who found striking similarity between pro-inflammatory cytokine expression patterns in bowel biopsies of PD patients and patients with inflammatory bowel diseases [19].

It has been postulated that the GI tract might be an entry route for a still unknown agens or neurotoxin that crosses the intestinal epithelial barrier, induces α-syn aggregation in the ENS and migrates retrogradely via projecting neurons towards the CNS [10]. A disturbed intestinal barrier function was recently observed in patients with PD [56]. In this context it is of note that increased mRNA levels of DRD1 and DRD2 in the intestinal mucosa after traumatic brain injury correlated with an impaired intestinal mucosal barrier function [73]. Thus, our observed upregulation of D1 receptor could point to a disturbed intestinal epithelial barrier in patients with PD. This hypothesis is in line with the impressive down-regulation of the muscarinergic M3 receptor, as M3 receptor is involved in the regulation of permeability in human jejunal epithelium and discussed as main mediator of transcellular transport of macromolecules [13]. Of note, M3R is known as an activator of cytoplasmatic phospholipase A2 via the activation of protein kinase c (PKC) [75] and since PKC itself has been shown to play a role in the modulation of tight junction proteins such as occludin [65], it is possible, that M3 receptors may be involved in the modulation of epithelial barrier permeability both via modulation of tight junction proteins and transcellular permeability in patients with PD.

### Correlation between p-α-syn positive aggregates and gene expression data

Correlation analysis between p-α-syn positive aggregates and gene expression data yielded a negative correlation between the expression level of the M3 receptor and the number of small sized p-α-syn positive aggregates, independent of both groups where correlation was lost when analysis was made separately. This suggested a direct link between p-α-syn assembling in submucosal neurons and transcript changing of M3R.

Positive correlation between mucosal α-syn staining and increased intestinal permeability or bacterial translocation in patients with PD was found before by *Forsyth et al*. [22]. Based on the widely accepted assumption that α-syn aggregates are a consequence of oxidative injury to neurons [62], the authors proposed that local oxidative stress caused by the translocation of luminal bacteria products leads to α-syn missfolding, aggregation and subsequent neuronal damage in the ENS. Since M3R expression may play a role in modulating the epithelial barrier, our observed direct negative correlation between M3R expression and small sized p-α-syn aggregates may further reflect a causality of increased intestinal permeability and α-syn assembling in patients with PD.

Interestingly, M3R activation is linked to α-syn in a human dopaminergic cell line in which muscarinic receptor stimulation leads to translocation of oligomeric α-syn from the plasma membrane to a light vesicle fraction in the cytoplasm [42]. The authors suggest, that α-syn could have a physiological role in the cell, in which its release transiently disinhibits membrane bound phospholipase LD2, freeing this lipase to function in ligand-stimulated manner to muscarinic receptor endocytosis. Our data therefore also suggest a physiological process of α-syn aggregation, which is linked to M3R receptor expression and therefore perhaps dysregulated in PD.

## Conclusions

In summary, we provide evidence that although suggested in previous studies, the mere presence of p-α-syn positive aggregates in the ENS cannot be regarded as a diagnostic criterion for PD, as p-α-syn is also consistently expressed in healthy controls. However, if a refined morphometric analysis is applied, a specific pattern of p-α-syn aggregates can be identified in patients with PD distinct from controls. Moreover, altered gene expression profiles observed in biopsies from patients with PD suggest that PD is associated with inflammatory processes and intestinal barrier dysfunctions possibly enhancing p-α-syn assembling. Further studies are needed to proof whether subtle quantitative and morphometric characterization of enteric p-α-syn may be an additional approach to allow diagnosis of PD by using GI biopsies.

## Additional files

Additional file 1: Figure S2. Mean ganglionic area, mean neuronal area and % p-a-syn positive aggregates per neuron of patients with PD and controls. Mean neuronal area (a), mean ganglionic area (b) and mean neuronal number per ganglionic area (c) exhibited no significant difference between patients with PD and controls. (TIF 2469 kb)
Additional file 2 Figure S1. UPDRS-III and Wexner constipation score. (a) The clinical semi-quantitative UPDRS-III test (motor symptom score: 0–108) yielded a mean score of 26.5 ± 16.19 in patients with PD and 0 in controls. (b) The Wexner constipation score (constipation symptoms: 0–20) yielded a mean score of 4.1 ± 5 in patients with PD and 1.6 ± 4.2 in controls. *n* = 10 controls, *n* = 12 patients with PD, *** *p* < 0.001 vs. controls. (TIF 996 kb)
