# Supplementary figures and images for: Distinct pattern of enteric phospho-alpha-synuclein aggregates and gene expression profiles in patients with Parkinson’s disease
Source: Acta Neuropathol Commun. 2017 Jan 5;5:1. doi: 10.1186/s40478-016-0408-2 (PMC5217296; doi:10.1186/s40478-016-0408-2)

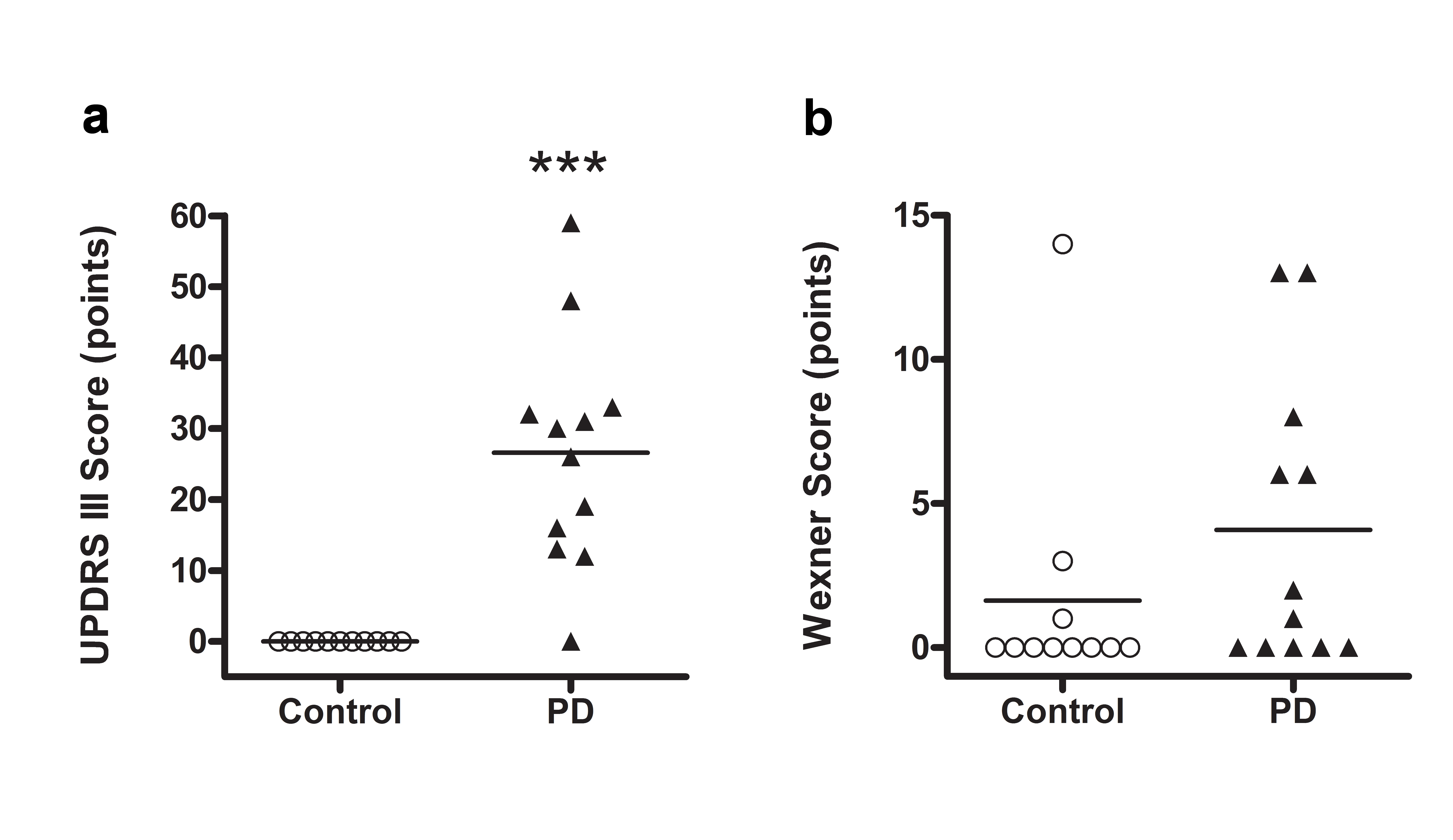

Supplement: Additional file 2 Figure S1. — UPDRS-III and Wexner constipation score. (a) The clinical semi-quantitative UPDRS-III test (motor symptom score: 0–108) yielded a mean score of 26.5 ± 16.19 in patients with PD and 0 in controls. (b) The Wexner constipation score (constipation symptoms: 0–20) yielded a mean score of 4.1 ± 5 in patients with PD and 1.6 ± 4.2 in controls. n = 10 controls, n = 12 patients with PD, *** p < 0.001 vs. controls. (TIF 996 kb) [file 40478_2016_408_MOESM2_ESM.tif]
